# Seeing the Forest for the Trees: How “One Health” Connects Humans, Animals, and Ecosystems

**DOI:** 10.1289/ehp.122-A122

**Published:** 2014-05-01

**Authors:** Wendee Nicole

**Affiliations:** Wendee Nicole was awarded the inaugural Mongabay Prize for Environmental Reporting in 2013. She writes for *Discover*, *Scientific American*, *National Wildlife*, and other magazines.

A gossamer mist settles over the jagged peaks of Bwindi Impenetrable National Park, a 318-square-kilometer park on the eastern flank of the Albertine Rift in southwest Uganda. It’s a hard scramble up and down steep ravines of this World Heritage Site,^1^ home to 400 of the world’s estimated 880 remaining mountain gorillas.^2^ The guide, Omax, radios ahead to trackers who have located the Habinyanja gorilla family. As the eight tourists and their porters catch up, everyone gathers to watch, mesmerized, as two gorillas placidly eat nettles. Without warning, a male gorilla named Kavuyo charges straight toward a middle-aged woman; she holds her ground, her eyes saucers. “He’s a joking one,” Omax says after shooing Kavuyo back.

**Figure:**
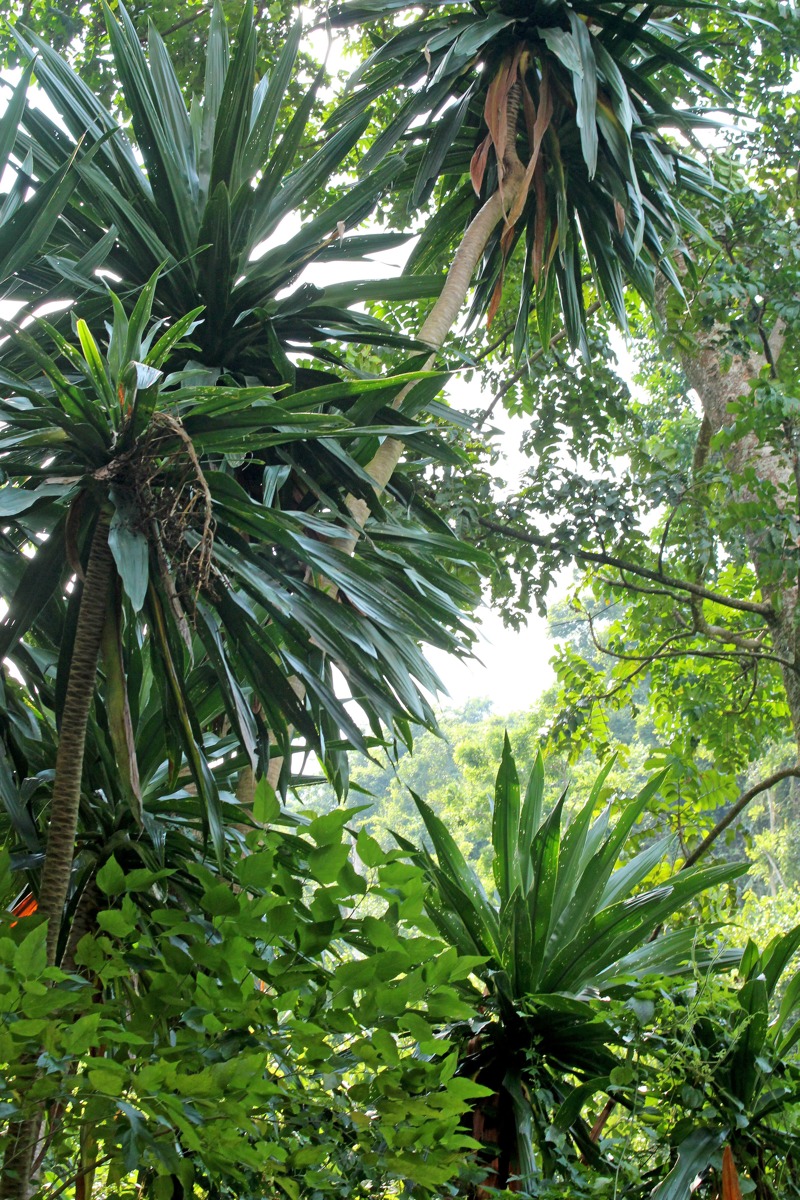
Writer Wendee Nicole traveled to Uganda to report this story under the Mongabay.org Special Reporting Initiatives Program. She tracked chimpanzees and mountain gorillas, and spent time with the Batwa people, “conservation refugees” living outside their former forest home. © Wendee Nicole

**Figure:**
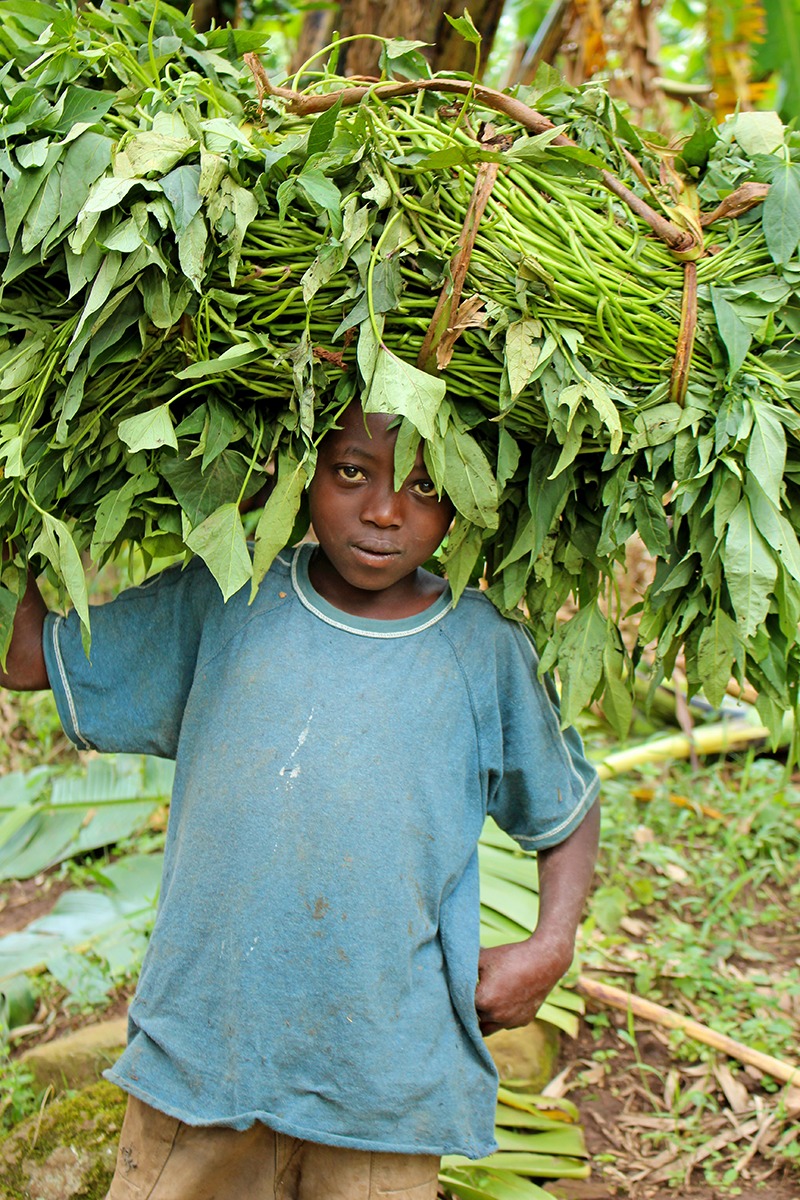
A Ugandan child collects sweet potato vines near Bwindi Impenetrable National Park. © Wendee Nicole

**Figure:**
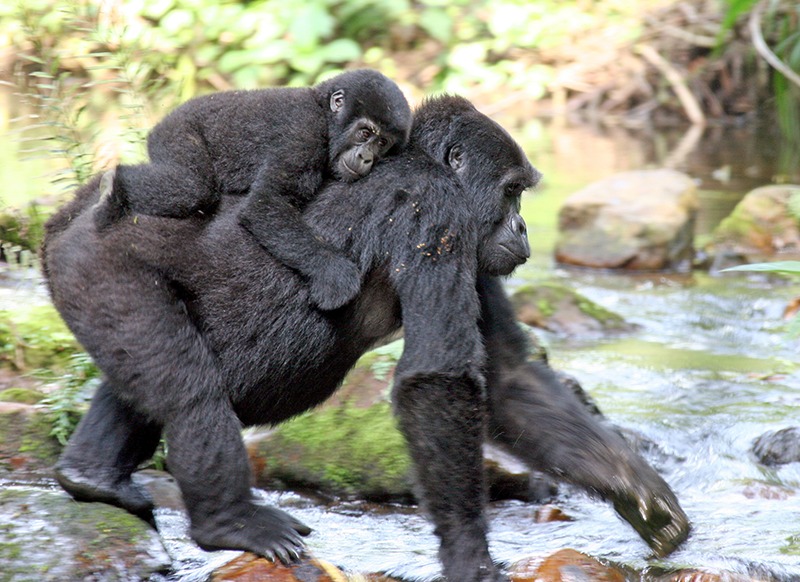
A mother gorilla and her baby cross a creek in the park. © 2014 The Kellermann Foundation. Used with permission.

Tourists have just one hour to watch the gorillas and must stay seven meters away from the animals, but counting tourists, porters, trackers, and guards, more than 60,000 people visit the park for the gorillas every year, in addition to locals passing through, potentially exposing both species to health hazards from the other.^3^ People and great apes are so closely related that infectious agents ranging from common cold viruses to potentially fatal diseases such as tuberculosis can pass between the two.^4^^,^^5^^,^^6^^,^^7^^,^^8^ One study found that 30% of park staff and 85% of local villagers admitted to defecating in the park without burying it, and many leave behind soiled trash that can expose the gorillas to parasites, pathogens, and other health threats.^3^

Outside the park, the potential health risks are even greater. Most families living and farming immediately outside the park do not have pit latrines, let alone flush toilets; 78% report defecating directly in their gardens, and 50% report using nearby bushes.^3^ When rains come, fecal matter left on the ground washes into waterways that livestock, wildlife, and people share for drinking and bathing.

Over the past two decades, the human population around the park has burgeoned, tourism has increased, and habituated gorillas have become less frightened of people. Human–wildlife conflict occurs when wild animals eat crops or damage property. Curious and enterprising, gorillas regularly raid people’s gardens^9^; for the poorest of the poor who live near the park boundary, this is a serious setback. This conflict can escalate poverty, increase disease spread, put people at risk from emerging zoonotic disease epidemics, and sometimes results in people killing or injuring wildlife^9^—a devastating consequence for critically endangered species such as mountain gorillas. In 1996 a gorilla infant died when its family contracted scabies mites, likely from curiously inspecting a villager’s scarecrow clad in mite-infested clothing.^10^ Scientists have documented other cases of infections likely passed from humans to gorillas.^11^

Recently, the dilemma of human–wildlife conflict has created great opportunity to solve some of the world’s most pressing problems for both people and ecosystems. The emerging “One Health” movement^12^ explicitly recognizes the inextricable connections between human, animal, and ecosystem health,^13^^,^^14^ and is leading not only to new scientific research but also to projects that help people rise out of poverty, improve their health, reduce conflicts with wildlife, and preserve ecosystems, such as Bwindi’s tropical montane forest.

In Africa and around the world, the integrated, holistic One Health effort has conservationists improving community health and people’s livelihoods, and health-care professionals participating in conservation.^15^ The authors of the Millennial Ecosystem Assessment have said that any hope of achieving the United Nations Millennium Development Goals^16^ not just for environmental sustainability but also for poverty eradication and improved health must explicitly consider the ecosystems that people depend on.^17^ The One Health approach shows promise for helping developing nations achieve these goals.^18^

## The History of One Health

The connection between animal and human health was recognized even in ancient times; later, nineteenth-century physician Rudolf Virchow coined the term “zoonosis,” writing that “between animal and human medicine there are no dividing lines—nor should there be.”^19^ In the late twentieth century epidemiologist Calvin Schwabe first proposed the idea of “One Medicine” encompassing both human and animal health.^20^ But medicine has since lost sight of the forest for the trees, now even to the point of focusing on individual leaves, says Laura Kahn, a physician and research scholar at the Woodrow Wilson School of Public and International Affairs at Princeton University.

“A schism has been developing in medicine for decades,” Kahn says: Should it focus strictly on individual care or more broad-based population-level health? Shortly after the anthrax attacks following 9/11, Kahn was reading the veterinary medicine literature and found herself struck by how many diseases of bioterrorism are—like anthrax—zoonotic. “Yet I discovered that [people working in] veterinary and human medicine and agriculture rarely talk to one another,” she says. “We’re trying to deal with new twenty-first-century challenges using outdated twentieth-century paradigms.”

With West Nile encephalitis, SARS, Ebola hemorrhagic fever, swine flu, and other zoonotic diseases popping up regularly in recent decades, scientists and medical practitioners have taken notice.^21^ In 2004 the Wildlife Conservation Society held the One World, One Health conference to bring together leaders from various disciplines; it culminated in the 12 Manhattan Principles, which urged world leaders, scientists, and society to more holistically consider the interrelationship between zoonotic diseases and ecosystems.^22^ Since then, more researchers have begun explicitly addressing how the dramatic changes happening to the Earth’s ecosystems affect human health.^23^ In 2008 Kahn cofounded the One Health Initiative website, a clearinghouse for news and publications related to the movement.^24^

Perhaps even more than in the United States, people living in developing countries recognize the value of a One Health approach. “The developing world sees the connections between human, animal, and environmental health more than the developed world does,” says Kahn. People still live with their livestock, they interact with wildlife more often, and they share common water sources with animals, among other issues. “There’s still open defecation; it’s shocking,” she says. “Today, we’re dealing with global population pressures, intensive agriculture, global trade and travel. All these things are taxing the ecosystems”—not to mention human livelihoods.

## A Hospital using a One Health Approach

When Bwindi Impenetrable National Park was formed in 1991, the Batwa people were evicted from their forest home; they became “conservation refugees,” and today most live in abject poverty around the park edges. U.S. missionary doctor Scott Kellermann arrived in Uganda in 2000 to survey the indigenous Batwa pygmies and found his calling. What started with Kellermann treating patients under a tree eventually became the Bwindi Community Hospital, which he says now boasts one of the most mature, comprehensive health outreach programs in sub-Saharan Africa—one that addresses the region’s poverty, health, and conservation ailments in a holistic way. “If you really want to help gorillas, if you believe there’s human–wildlife conflict, then what you do is improve people’s quality of life,” Kellermann says.

Originally just for the small Batwa population, the hospital now reaches more than 100,000 people per year in a 190-square-kilometer area. Scott and his wife Carol founded the Batwa Development Program (BDP) in 2008 to help the Batwa raise funds to support themselves. They do so by weaving baskets from local materials and teaching tourists—and their own children—their traditional ways with a cultural ecotourism program called the Batwa Experience. In February 2014 the Dalai Lama honored Scott and Carol with the Unsung Heroes of Compassion award.^25^^,^^26^

From the start, Kellermann understood that a hospital alone would not solve poverty and its associated health ills. He says, “It is commonly believed that hospitals improve the health of a population. This is not true. Hospitals typically treat only the sick; health care is improved only through preventive programs. Clean water, sanitation, food security, and access to health education improves health and reduces poverty.”

The Batwa have always treated their illnesses with medicinal herbs, hunted wild game, and harvested honey to survive. But when the park was first established, those activities became illegal; people lost a means of sustenance,^27^^,^^28^ and today, accessing forest products is prohibited without a permit.

**Figure:**
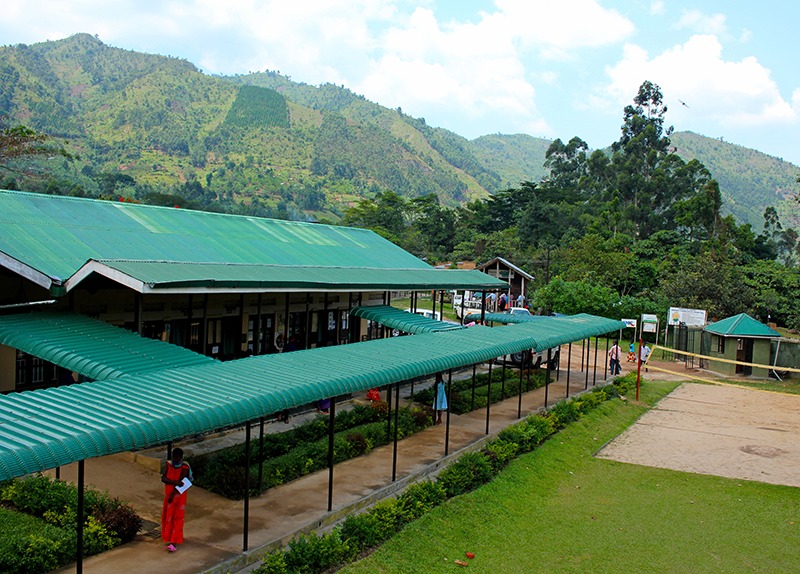
Bwindi Community Hospital, in the village of Buhoma just outside Bwindi Impenetrable National Park, serves more than 100,000 villagers in the surrounding area. Founder Scott Kellermann says the hospital’s outreach efforts address the region’s poverty, health, and conservation ailments in a holistic way. © Wendee Nicole

It takes time to change a culture; however, healthy people are less likely to access the forest for medicinal herbs or to poach wild animals for food, Kellermann says. If the Batwa received adequate health care and education—mosquito bed nets to prevent malaria, and information about the importance of hygiene and sanitation, for example—perhaps the reduced incidence of illness would mean less foraging for medicinal herbs. If they got adequate protein, perhaps they would not need to poach wildlife. Various organizations are continuing to address these issues.

Both the BDP and the hospital engage in weekly outreach to communities far and wide, not only collecting data on infectious diseases, births, and deaths, but also teaching people in their homes about health, hygiene, sanitation, and even conservation. “Educate the kids, particularly girls,” he says. “Girls attending school tend to have smaller family sizes, less HIV, less spousal abuse, and be more likely to advocate for their rights.”

In March 2014 the hospital sent four volunteers, including three Batwa women, to Tanzania to learn how to make fuel-efficient cook stoves that produce less smoke. “People do not know that pollution from firewood and open flames is hazardous. It is a silent killer,” explains Birungi Mutahunga, the hospital’s executive director. “[The volunteers] will be training the community to be able to make the stoves themselves and … that will minimize the need for people to go to the forest to get firewood, which brings people in contact with gorillas.”

**Figure:**
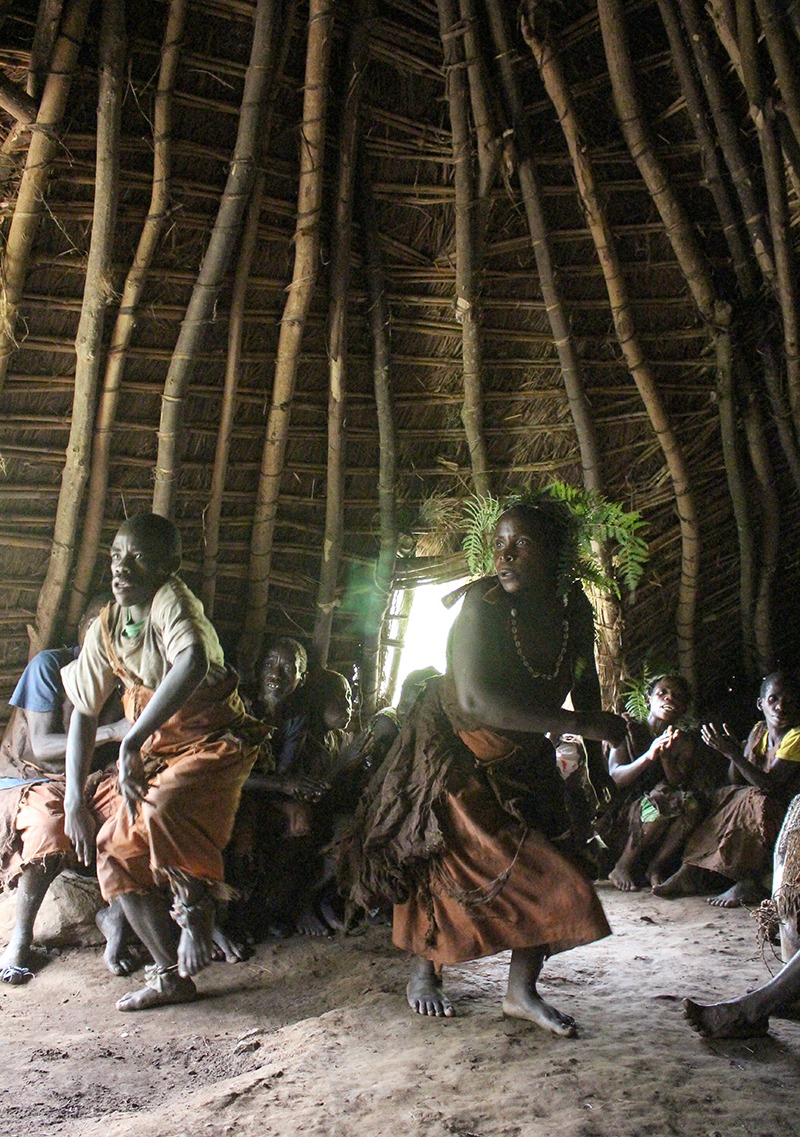
The Batwa are “conservation refugees,” evicted from their traditional home with the 1991 establishment of Bwindi Impenetrable National Park. Today the Batwa Experience ecotourism program enables the Batwa to pass their traditions along to younger generations and visitors, with the proceeds returning to Batwa communities. © Wendee Nicole

The hospital’s outreach program also teaches locals how to make “tippy taps,”^29^ converting water jugs and sticks into foot-operated hand-washing stations. Among nearby schools, the hospital increased the percentage of latrines with hand-washing facilities from 12% to 91% in just 12 months, and they also installed tippy taps in many homes. During the same period, there was a 50% decline in people admitted to the hospital for diarrheal diseases, says Mutahunga.

## An Economic Solution

Saving ecosystems while improving people’s livelihoods has been called a classic social–ecological dilemma, with the two outcomes typically at odds.^30^ Improving people’s health often means they lead longer lives and have more children, causing more degradation of stressed ecosystems. Likewise, conserving forests has often meant removing indigenous peoples or restricting local use of forest goods.

With that in mind, can a One Health approach really help people and ecosystems in the long run? Classic economic theory holds that people naturally act in “rational self-interest,” often contrary to the best interests of the larger group, in what Garrett Hardin in 1968 dubbed the “tragedy of the commons.”^31^ Many ecologists, economists, and policy makers have long assumed that the only way to protect natural resources is top-down ownership by a centralized government—create a national park, for example—or, at the opposite extreme, assign market values to ecosystem products or services.^32^

In the 1990s one optimistic political economist challenged the theory that people always act selfishly and would not work collaboratively to sustainably manage resources; Elinor Ostrom called these ideas “dangerous” when used unquestioningly as a foundation for policy. Ostrom, an Indiana University political science professor until her death in 2012, won the 2009 Nobel Prize in Economic Sciences for her paradigm-shifting work.^33^ After reviewing thousands of case studies and conducting her own research, Ostrom found that markets and states often failed to protect both ecosystems and human livelihoods. Instead, she found a third solution to solving this social–ecological dilemma: Give the local people most invested in using a common resource a say in its management.

Ostrom championed the idea that ordinary citizens can save ecosystems and improve human health and livelihoods, particularly if higher-level governments do not interfere with locally crafted arrangements. She identified several principles that make such situations successful, which included allowing the people using a common resource to make and modify the rules of use, making clear rules on who can and cannot use the resource, having outside authorities (local and national governments) respect local rules, ensuring that a monitoring system with appropriate sanctions is in place, and having cheap, accessible means of conflict resolution.^32^^,^^34^ “When people have the rights and freedoms to make their own decisions, it’s possible they do it a lot better than a government that’s centralized and doesn’t understand what it’s like on the ground,”^35^ says Catherine Tucker, an Indiana University associate professor of anthropology.

## Ostrom Applied

Recognizing the importance of giving more power to local authorities, many governments around the world, including Uganda, have formally adopted decentralization policies, allowing local and regional government entities to make more decisions.^36^^,^^37^ But they do not always pass this power along to local citizens.^38^ “What we see is increased interference with local arrangements, some of which have worked well for centuries or millennia,” Tucker says.

Ostrom found that evicting indigenous peoples or restricting resource use within a forest upon the creation of national parks often causes poaching and illegal harvest of forest products to increase rather than decline, creating a free-for-all because local rules, long established, get disrupted.^33^^,^^39^ When people suddenly have no rights to resources they previously could access, they have little motivation not to break the rules. In Bwindi, data suggest that poaching and forest product harvest have not declined since locals were restricted from these activities.^28^^,^^40^

In one study, Makerere University professor Abwoli Yabezi Banana, a regular scholar at Ostrom’s Workshop, compared five Ugandan forests managed in different ways. The one forest with Batwa living inside its borders experienced less illegal harvesting by locals, who were allowed to harvest forest products once per week.^41^ Banana’s study aligned with Ostrom’s principles, particularly that having locally vested forest monitors helps prevent a tragedy of the commons. Uganda’s government has started moving away from its initial strict “protectionist” policies in parks, allowing locals limited use of park resources, with mixed results.^28^^,^^36^^,^^42^ This has led to more positive views of the park by locals, but evidence suggests that the neediest and poorest citizens are not benefitting as much as others.^28^

**Figure:**
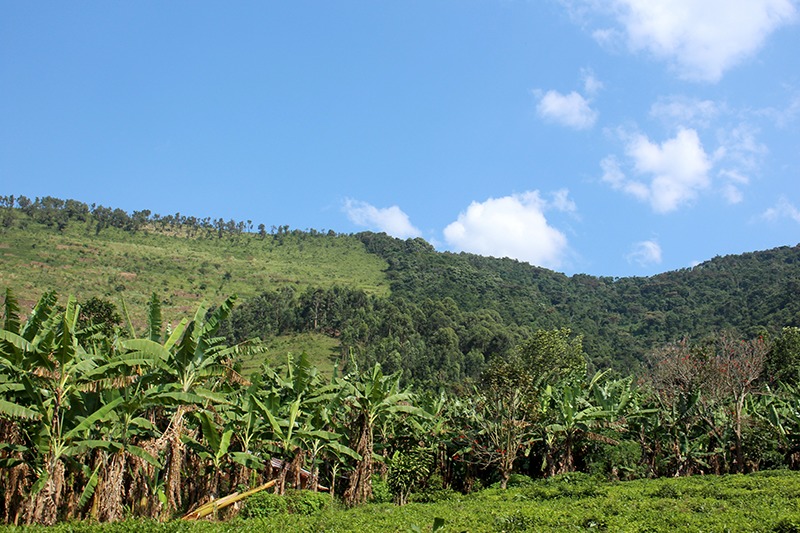
A hard edge exists between Bwindi Impenetrable National Park, home to the famous mountain gorillas, and the agricultural lands and homesteads surrounding it. © Wendee Nicole

Further north in Uganda, Tony Goldberg, an epidemiology professor at the University of Wisconsin–Madison, has applied Ostrom’s principles in a One Health framework in his work in and around Kibale National Park, a tropical forest and grassland overlooking the jagged Rwenzori Mountains. Outside the park, locals face similar human–wildlife conflict as the people near Bwindi, except with chimpanzees, elephants, and other wildlife that raid gardens. People nearby suffer from poverty and its associated health ailments.^43^

In his work as director of the Kibale EcoHealth Project, Goldberg has documented the interplay between the health of people and the health of ecosystems. “I’ve seen glaciers disappear [on the Rwenzoris]. I’ve seen forests become fragmented. I’ve watched human populations expand,” he says. “And we’re seeing a very clear effect on disease transmission and human health and animal health.”

The Kibale EcoHealth project has expanded beyond empirical research to implementing practical solutions to local problems. “When we talked to people in the community, the top concern was access to health care,” Goldberg says. So he partnered with colleagues from McGill University to build and run a medical clinic inside the park, a valuable service sanctioned by the Uganda Wildlife Authority. The team has gotten feedback from people who say it’s changed the way they view conservation.

“What we’re currently doing involves both ecological and social aspects, and is consistent with [Ostrom’s] overarching conclusions,” says Goldberg. “What I take away from her work … is that the solution needs to be engineered at the same scale as the problem. We work at the village level to solve village-level problems.”

## Sustainable Livelihoods

As Jane Goodall celebrates her 80th birthday this year, her legacy lives on in Africa. In west-central Uganda, the Jane Goodall Institute’s (JGI) Sustainable Livelihoods project not only aligns with the One Health perspective but also incorporates several of Ostrom’s principles. As human populations have expanded, chimp populations have declined throughout their range in central Africa—a classic social–ecological dilemma. Only 175,000 chimpanzees remain throughout their native range, with 5,000 remaining in Uganda.^44^ Meanwhile, Uganda’s human population grew from 8 million in 1962 to 34 million in 2012, with one of the world’s youngest populations (78% below age 30) and highest fertility rates (an average 6.4 children per woman).^45^

**Figure:**
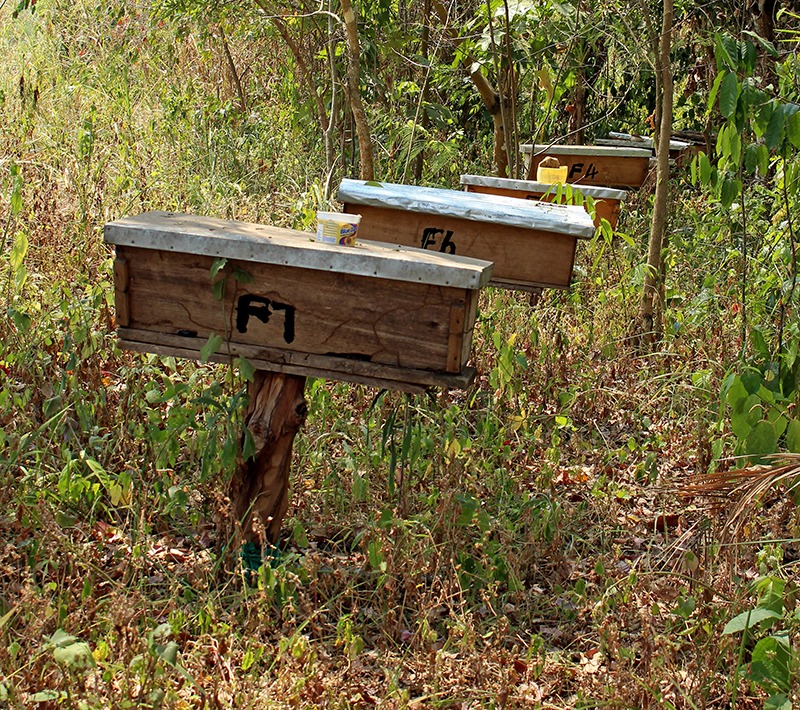
Some families participating in the JGI’s Sustainable Livelihoods project received beehives so they could produce and sell honey. This not only helps improve family incomes, but also reduces local residents’ need to illegally harvest honey from the forest. © Wendee Nicole

**Figure:**
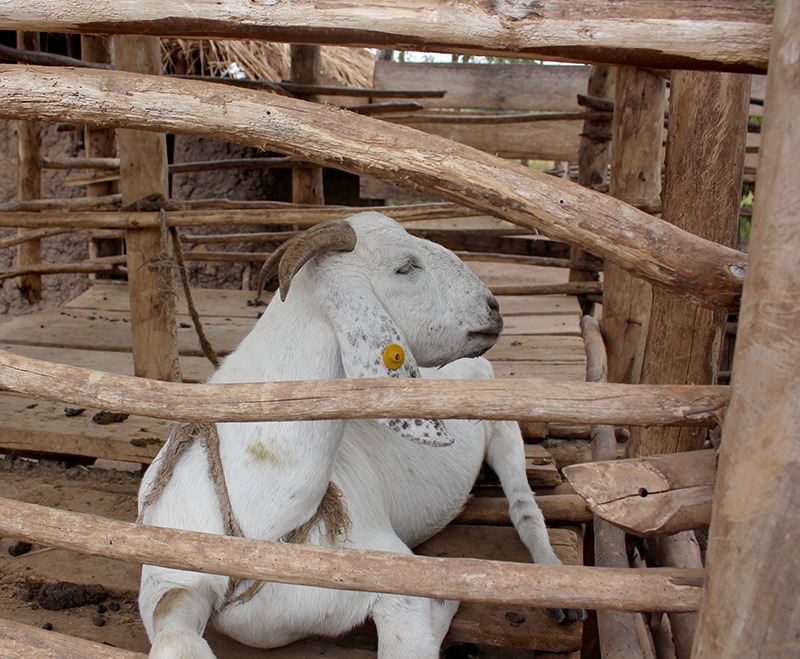
Other families received South African Boer goats, which grow larger and faster than local species. These goats also have a higher rate of twinning, and the offspring are shared with other families. © Wendee Nicole

“Because of the rapidly growing human populations, we’ve had a lot of fragmentation of what was, centuries ago, a continuous forest,” says Peter Apell, JGI’s field programs manager in Uganda. JGI wanted to reconnect two isolated chimp populations living in the Bugoma and Wambabya forest patches. “It was such a daunting task because connecting the fragments meant taking land away from communities that are living along that corridor,” Apell says.

The institute instead began working with the seven villages along the 6.4 kilometers of land connecting the forest patches. JGI staff met with community members and listened to their problems as well as their proposed solutions. “Many talked about how their level of poverty requires them to look for ways of improving their livelihoods,” Apell says. “A lot of them have said they are out hunting, they’re going into the forest to harvest wild honey, and they are facing problems because they get arrested. They say, ‘If I had money, I wouldn’t be hunting. If I had sheep or goats or pigs, I wouldn’t be hunting.’”

Even more than income and meat, the communities needed water. Rivers had dried up because locals farmed right to their edges, and siltation had filled them in. As a result, women and children walked for hours to gather water every day, sometimes causing children to miss school.^46^^,^^47^ JGI also noticed their agricultural practices were poor; for example they were using poor quality seeds, farming on steep slopes without terracing, and not rotating crops or properly mulching.

Not only did most villagers not believe that trees could restore the river or that new agricultural techniques would make a difference, they feared the government might take their land if forests and chimps returned, Apell says. JGI had to win over a skeptical crowd.

The institute began by improving goodwill; they installed one well per village, renovated five freshwater springs, and soon recruited a few pioneers. Participants received either improved crop seeds, beehives, Boer goats (which grow faster and larger than local goats), pigs, or training in basic forestry so they could raise tree seedlings for woodlots. Exotic fast-maturing species could be harvested for income, while indigenous trees would remain for a sustainable forest.

In exchange, JGI required participants to improve domestic hygiene and nutrition by undertaking a number of activities such as installing a pit latrine, establishing a kitchen garden, and constructing a drying rack to keep dishes off the bare ground. “These communities would wash their cups and plates using dirty water and then dry them out in the sun, like there, on the ground,” Apell says, pointing to the rich red soil. They also encouraged locals to build vented cook stoves to reduce smoke inhalation.

**Figure:**
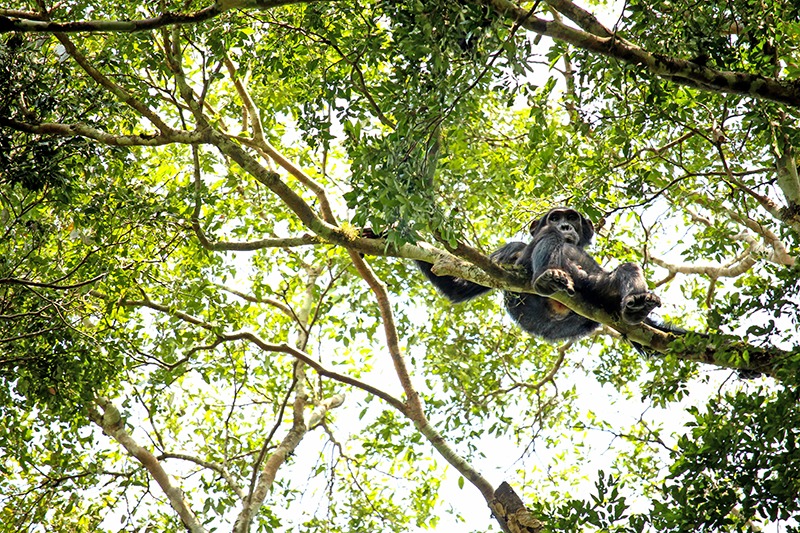
A chimpanzee relaxes in Budongo forest, a reserve with a relatively large population of the animals. JGI is promoting reforestation of other forests nearby in hopes chimps will return to those areas as well. © Wendee Nicole

The people saw that JGI’s concern was genuine, and participation grew rapidly. After seeing improved crop yields and increased household incomes from the few initial participants, soon everyone wanted to join, says Apell.

In line with Ostrom’s principles of following the locals’ lead in creating their own rules, JGI distanced itself from the decision-making process, but put in place the structure for the communities to lead the process themselves, according to Apell. The locals started a community association with representatives from each village. They elected a chairman and leaders, then divided themselves into interest groups—some groups wanted honey, some wanted trees, others wanted seeds.

Since JGI lacked funds to give animals or seeds to every family, they adopted the “pass on the gift” approach widely used by Heifer International, a project partner. When one family’s goat breeds, for instance, they pass a female kid to another family. Likewise with pigs and seeds. “Even after the project there are still people passing on goats to each other, passing on [seeds], passing on tree seedlings,” says Apell. The project was designed to be self-sustaining even after JGI’s involvement ended last year.

Although the new trees need to grow at least five more years before chimps return, 90% of the riparian forest has been restored, and black-and-white colobus monkeys have returned to the river corridor. “During the village implementation time, that area was more peaceful and less threatening [for wildlife],” says Apell. “Maybe the chimps are also watching.”

## Twenty-First-Century Challenges

Although Ostrom identified principles that help both forest health and livelihoods, she regularly stressed that no panaceas exist.^48^ But her legacy makes clear that, in order to see long-term success, whether in East Africa or around the world, One Health projects must explicitly account for the political, social, and economic settings in which the problems and projects occur.^49^ With projects ranging from conservation and public health initiatives being implemented on the ground to scientific research occurring around the globe, One Health shows much promise in creating holistic approaches to solving the world’s pressing—and interconnected—problems.

“People who promote global health need to realize you can’t have global human health without healthy livestock and wildlife. We don’t live in a vacuum,” says Kahn. “For the challenges we face in the twenty-first century, we need to be creative in confronting multidisciplinary threats. One Health is a creative, flexible concept that promotes interdisciplinary thinking and collaboration.”
